# Including glutamine in a resource allocation model of energy metabolism in cancer and yeast cells

**DOI:** 10.1038/s41540-024-00393-x

**Published:** 2024-07-18

**Authors:** Jan Ewald, Ziyang He, Wassili Dimitriew, Stefan Schuster

**Affiliations:** 1 Department of Bioinformatics, Friedrich Schiller University of Jena, Ernst-Abbe-Platz 2, 07743 Jena, Germany; 2 Center for Scalable Data Analytics and Artificial Intelligence (ScaDS.AI) Dresden/Leipzig, Leipzig University, Humboldtstraße 25, 04105 Leipzig, Germany

**Keywords:** Biochemical networks, Computer modelling, Cancer, Microbiology

## Abstract

Energy metabolism is crucial for all living cells, especially during fast growth or stress scenarios. Many cancer and activated immune cells (Warburg effect) or yeasts (Crabtree effect) mostly rely on aerobic glucose fermentation leading to lactate or ethanol, respectively, to generate ATP. In recent years, several mathematical models have been proposed to explain the Warburg effect on theoretical grounds. Besides glucose, glutamine is a very important substrate for eukaryotic cells—not only for biosynthesis, but also for energy metabolism. Here, we present a minimal constraint-based stoichiometric model for explaining both the classical Warburg effect and the experimentally observed respirofermentation of glutamine (WarburQ effect). We consider glucose and glutamine respiration as well as the respective fermentation pathways. Our resource allocation model calculates the ATP production rate, taking into account enzyme masses and, therefore, pathway costs. While our calculation predicts glucose fermentation to be a superior energy-generating pathway in human cells, different enzyme characteristics in yeasts reduce this advantage, in some cases to such an extent that glucose respiration is preferred. The latter is observed for the fungal pathogen *Candida albicans*, which is a known Crabtree-negative yeast. Further, optimization results show that glutamine is a valuable energy source and important substrate under glucose limitation, in addition to its role as a carbon and nitrogen source of biomass in eukaryotic cells. In conclusion, our model provides insights that glutamine is an underestimated fuel for eukaryotic cells during fast growth and infection scenarios and explains well the observed parallel respirofermentation of glucose and glutamine in several cell types.

## Introduction

Understanding cellular metabolism is a fundamental step to develop therapies for many diseases. Especially in complex diseases like cancer or infections, where invading and colonizing cells overwhelm the host, an altered metabolism is crucial for cancer growth^1–4^ and pathogenesis of microorganisms^5,6^. On the other hand, immune cells also activate their metabolism when they encounter cancer or pathogen cells^7,8^. Apart from specific disease-associated pathways, energy metabolism provides the fuel for all other processes and is a key target of cancer therapies and infection control^2,3,6,9^.

A common metabolic response of eukaryotic cells in stressful conditions or fast growth, which both require fast energy supply, is a switch from glucose respiration to fermentation or respirofermentation even under sufficient oxygen supply^10–12^. In human cells, this was first described by Otto Warburg in cancer cells^13^. It is worth mentioning that both glucose fermentation and respiration are important for cancer metabolism^14–16^. The term Warburg effect is also used for similar observations in other human cell types like activated lymphocytes and microglia cells^10,11^. A similar phenomenon in the baker’s yeast *Saccharomyces cerevisiae* was described and named the Crabtree effect after biochemist Herbert G. Crabtree^17^. It implies a downregulation of oxidative phosphorylation. Interestingly, pathogenic yeasts like *Candida albicans* prefer glucose respiration (and are therefore Crabtree-negative)^18,19^, which raises questions about the evolution and trade-offs underlying this observation. It was found that *C. albicans* mainly uses glucose fermentation and respiration during the lag phase and exponential growth phase, respectively^20^, which indicates that fast growth of this fungus requires respiration, in contrast to the Crabtree-positive *S. cerevisiae* and cancer cells. This is also supported by the observed reduction in growth rate (20 to 25%) when respiration via the electron transport chain (ETC) was eliminated^21^. It is worth noting that the ETC is not the only way for *C. albicans* to respire (albeit the most important), the second being a shortcut of the ETC from ubiquinone to oxygen via alternative oxidases. There are different opinions in the literature on whether a reduction in respiration in *C. albicans* stimulates the transition from yeast to hyphae^22–28^. It seems that the answer strongly depends on experimental conditions. As this effect is not very clear, we will not consider it in our minimal models.

In Table 1 we provide an overview of selected eukaryotic cells and their characteristics, requirements and observed metabolic modes. It exemplifies that the observed energy metabolism is linked to growth, energy or biomass demands, enzyme characteristics, and substrate availability. Although we consider neither macrophages nor muscle cells in our study, we have included them in the table to extend the overview.

**Table 1:** Overview of selected eukaryotic cells and their characteristic features of energy metabolism

	Human			Yeasts	
	Cancer cell	Macrophage	Muscle cell	*S. cerevisiae*	*C. albicans*
Proliferation and doubling times	varying, typically 20−60 h^81^	stimulated cells: 20−40 h, resident or phagocytosing cells are mostly non-proliferative^44,82,83^	originating from proliferating progenitors^84^	ideal conditions: 1.5 h^85^, barley malt: 5 h^86^	ideal conditions: 1 h^87^, non-proliferative inside macrophages^44^
Process requiring additional biomass or energy		cytokine and ROS production upon activation^83^	muscle contraction^88^		hyphae formation^18,89^
Glucose availability	via blood, 3.5−5.5 mM^37^			sugar >250 mM during ethanol production	not quantified in phagolysosomes; via blood during invasive growth
Glutamine availability	via blood, 2.5 mM^38^			not a primary source during ethanol production	not quantified in phagolysosomes; via blood during invasive growth
Observed energy metabolism	Warburg and WarburQ effects^34,36^	Warburg effect upon activation^90^	aerobic glycolysis during heavy exercise^88^	Crabtree effect^17^	Crabtree-negative^19^

Next to glucose, the uptake of glutamine is frequently observed in cells with high energy or biomass demand^29–32^. In addition to its function as a nitrogen source, glutamine can be converted to pyruvate in eukaryotic cells in a process termed glutaminolysis in analogy to glycolysis^33,34^. The process of glutamine respirofermentation, which was named WarburQ effect in analogy to the respirofermentation of glucose^35^, was primarily described for tumor cells and can drive biosynthesis and energy generation for cellular growth^34,36^. Tumor cells, immune cells but also microorganisms entering the bloodstream are supplied with high concentrations of glucose (3.5–5.5 mM)^37^ and glutamine (2.5 mM), the most abundant amino acid in the plasma^38^. It is worth noticing that, unlike *S. cerevisiae*, *C. albicans* can grow using amino acids as a sole carbon source^20^.

Since eukaryotic cells, especially in the human body, have multiple options regarding carbon sources and metabolic routes, many approaches were used to understand the evolution and trade-offs leading to the differences in energy metabolism among cells^39–42^. In the light of evolution, the concept of optimality is powerful in explaining the properties of living organisms^43–45^. However, standard Flux Balance Analysis (FBA) assuming yield maximization fails in elucidating the Warburg and Crabtree effects because respiration (allowing a higher ATP yield) rather than fermentation would be obtained unless an upper bound on oxygen consumption is included as an additional side constraint^46,47^. It is worth noting that the Warburg effect occurs even if sufficient oxygen is available^48^.

In contrast, to properly describe the Warburg and WarburQ effects, the reallocation of protein among enzymes is of importance. This can be modeled by resource allocation models, which use a constraint for a linear combination of velocities. By this extension to standard FBA, one can obtain results in agreement with the experimental data^10,41,42,49–53^. A particular approach is called FBA with macromolecular crowding (FBAwmc); the aforementioned constraint then concerns the crowding of enzymes in the intracellular space^41^ or within membranes^54,55^. Ranging from minimal models^51^ to genome-scale models^42^, these computational approaches lead to the insight that the Warburg effect can be understood in terms of rate maximization or enzyme cost minimization. In contrast, respiration provides maximal yield (rather than rate) of ATP per mole of glucose, which may not be accompanied by fast growth. While a few models of glutamine respiration and/or fermentation have been proposed^35,42,56^, most models of the Warburg effect focus on glucose utilization (see references above).

**Fig. 1:**
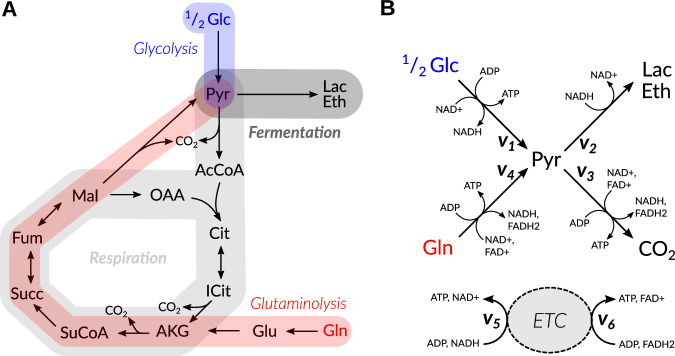
Metabolic routes and simplified model of energy metabolism. **A** The substrates glucose (Glc) and glutamine (Gln) can both be respired to CO_2_ or fermented to lactate (Lac) in humans or ethanol (Eth) in *S. cerevisiae* and *C. albicans*^19^^,91^ via different routes (colored and shaded boxes). **B** Pyruvate (Pyr) is used in our model as a hub between the different metabolic routes. Overall, reactions 1–4 describe lumped pathways which generate energy via ATP and the cofactors NADH or FADH_2_. The conversion of NADH or FADH_2_ to ATP is described by reactions 5,6. *v*_1_ – *v*_6_, fluxes through reactions 1–6 at steady state.

Here, we propose a minimal model for the fermentation and/or respiration of glucose and/or glutamine. It is an extension of an earlier minimal model describing the Warburg effect^51^, in which only glucose was considered as a substrate. Throughout the manuscript, we will use the terminology of reactions as shown in Fig. 1 and refer to the catabolism of glucose or glutamine leading to pyruvate as glycolysis or glutaminolysis, respectively. We compare the results for human cells with those for fungal cells to understand the different cellular behaviors of eukaryotes during energy-demanding scenarios like rapid growth or infection.

## Results

### Linear optimization reflects observed energy metabolism

**Fig. 2:**
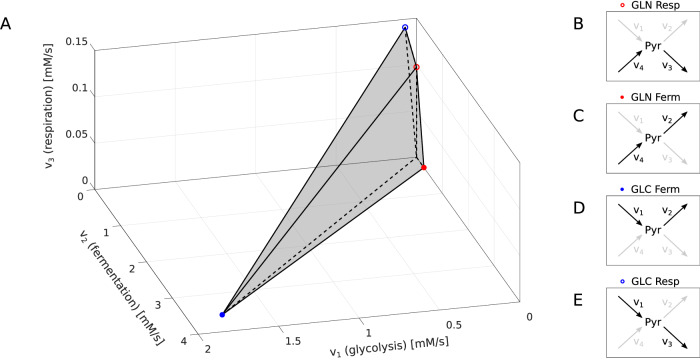
Solutions of linear optimization model of energy metabolism including glutamine. **A** Solution space spanned by the fluxes through the model’s reactions, with the parameter values for a human cell model. Due to the steady-state condition, the solution space is uniquely described by the fluxes *v*_1–3_. All constraints form the polyhedron of the solution space (gray), and pure metabolic routes are represented by colored vertices: glucose (GLC) respiration or fermentation (open or filled blue circle, respectively) as well as glutamine (GLN) respiration or fermentation (open or filled red circle, respectively). Coordinates *v*_1_, *v*_2_, *v*_3_ of the vertices in the order given above [0.0707, 0, 0.1414], [1.7804, 3.5607, 0], [0, 0, 0.0972], [0, 0.2863, 0]; see also Table 2. Note that the origin of the coordinates is located at the rear bottom right corner. **B**–**E** Elementary modes in our model that correspond to the polyhedron’s vertices.

The linear optimization problem described in the Methods section (see below) can be solved by calculating the objective function values at the outer vertices of the feasible region. Without a constraint on uptake rates, these vertices represent elementary flux modes^57^. In our model we have four elementary modes, which describe pure glutamine fermentation or respiration and pure glucose fermentation or respiration (see Figs. 1B, 2B–E and Table 2). Each of them only uses two fluxes from *v*_1−4_ and do not include the other two.

**Table 2:** General solution of the linear optimization problem by coordinates and objective function values of vertices (metabolic routes) describing the feasible regions

Metabolic route	Coordinates (*v*_1_, *v*_2_, *v*_3_)	Objective value
Glutamine respiration	$\left(0,0,\frac{1}{{\alpha }_{3}\,+\,{\alpha }_{4}\,+\,7{\alpha }_{5}\,+\,2{\alpha }_{6}}\right)$	$\frac{{m}_{3}\,+\,{m}_{4}\,+\,7{m}_{5}\,+\,2{m}_{6}}{{\alpha }_{3}\,+\,{\alpha }_{4}\,+\,7{\alpha }_{5}\,+\,2{\alpha }_{6}}$
Glutamine fermentation	$\left(0,\frac{1}{{\alpha }_{2}\,+\,{\alpha }_{4}\,+\,2{\alpha }_{5}\,+\,{\alpha }_{6}},0\right)$	$\frac{{m}_{2}\,+\,{m}_{4}\,+\,2{m}_{5}\,+\,{m}_{6}}{{\alpha }_{2}\,+\,{\alpha }_{4}\,+\,2{\alpha }_{5}\,+\,{\alpha }_{6}}$
Glucose fermentation	$\left(\frac{1}{{\alpha }_{1}\,+\,2{\alpha }_{2}},\frac{2}{{\alpha }_{1}\,+\,2{\alpha }_{2}},0\right)$	$\frac{{m}_{1}\,+\,2{m}_{2}}{{\alpha }_{1}\,+\,2{\alpha }_{2}}$
Glucose respiration	$\left(\frac{1}{{\alpha }_{1}\,+\,2{\alpha }_{3}\,+\,10{\alpha }_{5}\,+\,2{\alpha }_{6}},0,\frac{2}{{\alpha }_{1}\,+\,2{\alpha }_{3}\,+\,10{\alpha }_{5}\,+\,2{\alpha }_{6}}\right)$	$\frac{{m}_{1}\,+\,2{m}_{3}\,+\,10{m}_{5}\,+\,2{m}_{6}}{{\alpha }_{1}\,+\,2{\alpha }_{3}\,+\,10{\alpha }_{5}\,+\,2{\alpha }_{6}}$

The cost parameter *C* is normalized to be unity. For simplicity’s sake, the subscript ATP in the stoichiometric coefficients was omitted.

Since the molecular weight of enzymes reflects a good estimator for enzyme cost^45,58^ and is easily calculable, we carefully collected the enzyme mass per active site for each enzyme along the reactions *v*_1−6_ (see Supplementary information). The general solution based on the parameters is given in Table 2 and shows that the optimal metabolic route is dependent on the cost of the corresponding enzymes (reflected by weights *α*_1−6_) and the ATP stoichiometry (reflected by coefficients *m*_1−6_). For describing the Warburg and WarburQ effects, the relative flux distributions between fermentation and respiration rather than absolute values are of importance. Therefore, the total capacity *C* can be normalized to be one.

**Fig. 3:**
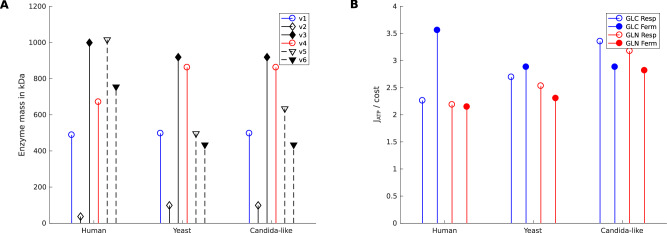
Cross-species comparison of the particular routes of energy metabolism. Colors indicate the substrates of the lumped reactions (blue, glucose; red, glutamine; empty circles, respiration; full circles, fermentation). **A** For each lumped reaction, the sums of the molecular weights per catalytic center of all enzymes are visualized and allow a comparison of the related costs in the human, baker’s yeast, and *C. albicans* models. **B** The costs from (**A**) are used to calculate the ATP rate per MDa of molecular enzyme mass for the four possible elementary modes. Note that not all of the fluxes need to be active in the optimal state. Parameter values: Supplementary Tables 1, 2; *C* = 1.

The required enzyme mass per active site and reaction step is around 50−100 kDa for most cytosolic enzymes and considerably higher for enzyme complexes of the ETC, notably around 200 kDa per active site and reaction step. Since pyruvate fermentation requires only one (to lactate) or two (to ethanol) reaction steps, respiration of glucose or glutamine requires much more enzyme mass (see Fig. 3A). While in general, yeast enzymes have similar (slightly smaller) molecular weights as their human homologs, the ETC complexes of yeast are significantly smaller due to non-homologous enzymes like complex I substituents (see Fig. 3A and cf. Supplementary information). In contrast, glutamine assimilation in yeast requires an alternative pathway, namely the GS-GOGAT cycle^59^, with higher enzyme masses than the human variant. All of these factors, together with the different ATP yield per NADH, influence the optimal metabolic route to maximize the ATP rate per enzyme mass. In both human and *S. cerevisiae* cells, glucose fermentation is superior over glutamine as a substrate and over respiration—as a metabolic mode (see Fig. 3B). The superiority of glucose fermentation over glucose respiration is the classical Warburg effect. Due to the smaller enzymes of the ETC, the difference between glucose fermentation and other metabolic routes is smaller in the yeast models than in the human cell model. Interestingly, the usage of a high-yield complex I (as in the Crabtree-negative *C. albicans*) instead of the low-yield complex I analog (as in *S. cerevisiae*) leads to glucose respiration as the optimal metabolic route for maximal ATP generation (see Fig. 3B). Accordingly, the optimal solutions for the human cancer model and *Candida* model are reached at the blue dot and blue circle, respectively, in Fig. 2A. Summarizing the above, the model reflects well the metabolic behavior of the eukaryotic cells and confirms glucose to be the primary source for energy metabolism. However, glutamine respiration, as well as fermentation, generates comparable amounts of ATP, especially in the *Candida* model, and can substitute glucose under limiting scenarios as elaborated in the next section (see Fig. 3B).

### Flux constraints cause mixed energy metabolism

In many situations, fluxes through the reactions of energy metabolism in human cancer (or immune) cells and yeast cells are limited. The supply of the carbon sources glucose (*v*_1_) or glutamine (*v*_4_) is high but certainly limited for cancer cells or pathogenic yeasts like *C. albicans*. When *C. albicans* cells are engulfed by macrophages, their glucose supply is limited, so that they switch to fatty acid consumption. Inside non-vascularized tumors, nutrients and oxygen are scarce. Fluxes through fermentation or respiration (reactions 2 and 3, respectively) may reach their maximal capacity due to the lack of sufficient enzymes, cofactors, or oxygen.

**Fig. 4:**
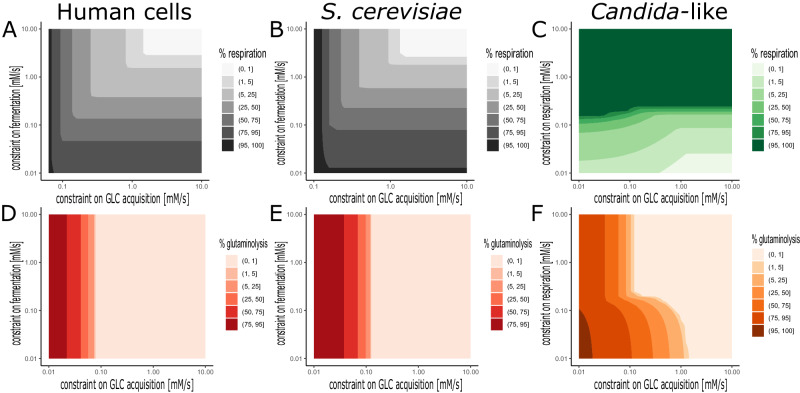
Heat map showing the optimal flux allocations for different scenarios. In **A**–**C**, the optimal flux allocation between *v*_2_ (fermentation) and *v*_3_ (respiration) is shown as *v*_3_/(*v*_2_ + *v*_3_), in %, in human, *S. cerevisiae,* and *Candida*-like models, respectively. Analogously, **D**–**F** show the optimal flux allocation between *v*_1_ (glucose acquisition) and *v*_4_ (glutamine acquisition) as *v*_4_/(*v*_1_ + *v*_4_), in %, in the corresponding models. For human and *S. cerevisiae* models, the optimal flux allocations are shown as functions of maximal fluxes through reactions 1 and 2 (*v*_1*cap*_, *v*_2*cap*_); for the *Candida*-like model, as functions of maximal fluxes through reactions 1 and 3 (*v*_1*cap*_, *v*_3*cap*_).

We simulate this by maximal velocity constraints and a recalculation of the optimal flux distribution in the energy metabolism based on our simplistic model. Since human (cancer) cells and *S. cerevisiae* show a preference for glucose fermentation^48,60^, we analyzed the influence of reduced capacities on the fluxes through the glycolysis and fermentation reactions 1 and 2 (Fig. 4). In contrast, in *Candida*-like species with a high-yield complex I, glucose respiration showed the highest ATP flux. Hence, for these species, we simulate a capacity limit on fluxes through the lumped glycolysis and respiration reactions 1 and 3.

**Fig. 5:**
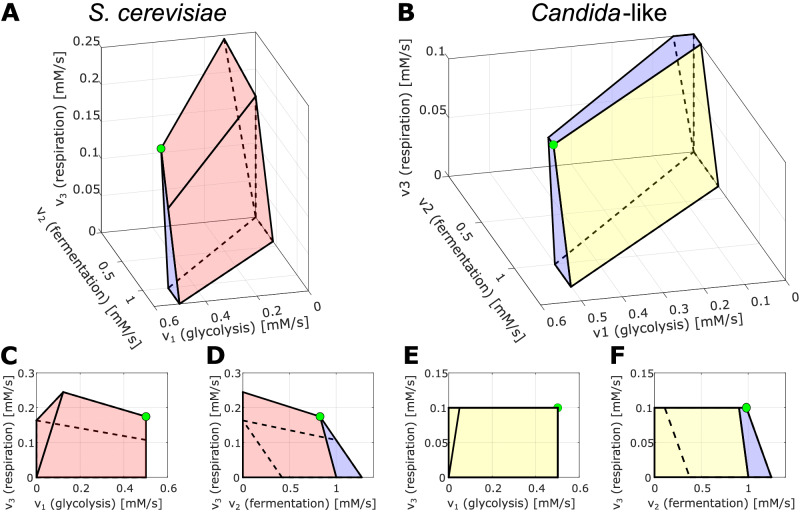
Solution spaces spanned by the fluxes through the model’s reactions, analogous to the one presented in Fig. 2A, for two other models. Due to the steady-state condition, the solution space is uniquely described by the fluxes *v*_1−3_. **A** *S. cerivisiae* model and the case of limitation of *v*_1_. The flux *v*_4_, which is not shown in the plot, is equal to zero in the optimal state - therefore, the optimal strategy for this model is glucose respirofermentation. **B** *Candida* model and the case of limitation of *v*_1_ and *v*_3_. The flux *v*_4_ (not shown) is positive in the optimal state. Therefore the optimal solution is the respirofermentation of both glucose and glutamine. Faces of the initial polyhedron - red (**A**), yellow (**B**); blue - face/s caused by the limitation of *v*_1_ (**A**), *v*_1_ and *v*_3_ (**B**); green dot, solution of the optimization problem. **C**, **E** Projection of the polyhedron onto the *v*_1_-*v*_3_ plane. **D**, **F** Projection of the polyhedron onto the *v*_2_-*v*_3_ plane.

A reduced glucose supply or fermentation capacity leads, in both human and yeast cells, to a mixed respirofermentation as the optimal energy metabolism, where the fraction of respiration is determined by the flux capacity constraints that limit *v*_1_ (e.g., the capacity of glucose transporters) and *v*_2_ and the constraint on enzyme costs (see Fig. 4A, B). Further, the usage of glutamine is affected by the constraint on glucose supply (*v*_1_), but not by a reduced fermentation capacity (*v*_2_, see Fig. 4D, E). This means that, in the case of human and *S. cerevisiae* models, reducing fermentation does not force cells to use glutamine (see also Fig. 5A). In other words, the importance of glutamine uptake for those cells is not confirmed by our model of energy metabolism; it is suggested that glutamine more likely serves biomass synthesis requirements.

In the *Candida*-like cells with a preference for glucose respiration, the influence of capacity constraints along the metabolic route (*v*_1_, *v*_3_) is different from the one in human and *S. cerevisiae* cells. The fraction of respiration appears to decrease when respiratory capacity is lowered under a certain threshold, but shows also a nonlinear and step-wise influence by glucose supply limitation, favoring more fermentation (see Fig. 4C). In a similar way, glutamine usage increases as glucose supply is more and more limited; the same behavior is observed in case of the limitation of respiration provided that glucose is also limited (see Fig. 4F), indicating a more pervasive importance of glutamine in the energy metabolism of *Candida spp*. The solution space for the *Candida* model with the limitation for *v*_1_ and *v*_3_ is shown in Fig. 5B; the optimal strategy is, as mentioned before, the combination of respiration and fermentation, utilizing both glucose and glutamine (see Table 3). The solution for baker’s yeast with the limitation of *v*_1_ is respirofermentation as well, yet without glutamine consumption (*v*_4_ = 0, Fig. 5A and Table 3).

**Table 3:** Flux values of the optimal solutions shown in Fig. 5

Flux	*S. cerevisiae*	*Candida*-like
*v*_1_	0.5012	0.5012
*v*_2_	0.8277	0.9812
*v*_3_	0.1747	0.1000
*v*_4_	0	0.0789

## Discussion

Many experimental and theoretical studies investigated the Warburg and Crabtree effects. There is evidence that many cancer hallmarks are the results of the Warburg effect^61^. Recently, the utilization of glutamine by cancer cells has aroused more and more attention^34,36,62^. While glutaminolysis is extensively studied experimentally, conclusive theoretical models for explaining the utilization of glutamine in terms of fast and efficient growth of cancer cells are still scarce.

To reach a better understanding of the energy metabolism in human cells, we extended a minimal model of the Warburg effect to study, in addition, the usage of glutamine via fermentation, which is related to the WarburQ effect, and via respiration. This led to a minimal model of this effect, since it involves six reactions only. In contrast, earlier models of this effect involve more than 70 reactions^35,56^ or are even genome-scale^42^. Like in the earlier three-reaction model^51^, the inclusion of an upper limit on the uptake of glucose allows us to model the respirofermentation of this substrate (Warburg and Crabtree effects). It is worth mentioning that Otto Warburg himself described a mixture of glucose fermentation and respiration^48^.

In our model, the reaction rates are considered as the variables of the optimization problem, without making any assumptions regarding rate laws. The rates are considered to be proportional to the enzyme concentrations, so that the side constraints can be written in terms of the rates. In more detailed approaches, the interrelation between the rates due to utilization of common metabolites such as pyruvate was taken into account^50,63^. Since enzyme kinetic rate laws are nonlinear, the side constraints are nonlinear in terms of metabolite concentrations even if they are linear in terms of enzyme concentrations. It was shown that the optimal solutions in any metabolic network correspond to elementary modes^50,63^, like in our simplified approach.

By parameterization we adapted our linear model also to *S. cerevisiae* and fungal pathogens like *C. albicans* to understand the role of glutamine in comparison to glucose in energy metabolism of other eukaryotes and infection scenarios. As it was mentioned above, *C. albicans* can grow solely on amino acids and has access during the bloodstream infection to both glucose and glutamine. Baker’s yeast can utilize glutamine as a (sole) nitrogen source^64,65^, but not as a sole carbon source^66^. Moreover, *S. cerevisiae* cannot use amino acids in general as a sole carbon source^66^, and does not use glutamine as a carbon source when glucose is available^65^. Glutamine fermentation possibly happens during wine fermentation, which leads to faster fermentation and promotion of volatile compound formation^67^. Glutamine, as well as another good nitrogen source, ammonia, represses many genes connected with nitrogen metabolism^65^.

Our results for human and baker’s yeast models indicate and confirm glucose as the preferred substrate and fermentation as the preferred route for cost-efficient generation of energy in the form of ATP. It seems reasonable to also consider the fact that proliferating cells need carbon atoms, and it should not be rational to waste all of them as CO_2_ during respiration^11,68^. Interestingly, the aforementioned strategy does hold true for human cells and *S. cerevisiae* but not for our model of the fungal pathogen *C. albicans*, where glucose respiration is superior over fermentation. This is in line with experimental findings that *C. albicans* is Crabtree-negative and primarily uses respiration during fast growth phases^19^.

In addition to the analysis of glutamine utilization, we also explained the observation that *C. albicans* prefers glucose respiration over glucose fermentation (i.e., Crabtree-negative), by the fact that this fungus has a complex I different from the one in baker’s yeast and several enzymes different from those in humans with respect to their molecular masses. These differences can be captured by our minimal model. It is an interesting question why in *S. cerevisiae* (in comparison to *C. albicans*), the typical complex I is substituted by an enzyme that does not pump protons, so that the ATP yield is lower than in higher animals, for example. This can be regarded as a trade-off between a high-yield/low-rate pathway (e.g. respiration using complex I) and a low-yield/high-rate pathway (e.g., fermentation), which can even be adjusted to varying environmental conditions^39^.

Our results support the finding that glutamine is a valuable resource fueling fast tumor growth^36^. In addition to cancer metabolism, the results also have implications for infection scenarios and host-pathogen interaction. Glutamine has a high blood concentration^38^, albeit lower than glucose concentration^69^; our optimization results indicate that under limited glucose and fermentation scenarios, glutamine is a comparably efficient substrate. Further, our results show that glutamine respiration and glutamine fermentation have a comparable ATP rate per enzyme mass, which is, moreover, nearly identical to the one of glucose respiration. While it is inferior to glucose fermentation in the human and baker’s yeast model, glutamine respiration even surpasses glucose fermentation in the *Candida*-like model. This supports the experimental finding and the view that, in cancer cells, glucose is fermented to lactate to generate energy, while glutamine respiration is used in addition to fuel the growth of cancer cells and biomass generation^70^.

Our results show that when no constraint on substrate uptake is included, the optimal flux distributions coincide with elementary modes, in agreement with earlier theoretical findings^40,50,63^. In contrast, when glucose uptake is considered to be significantly limited, a superposition of fermentation and respiration is obtained.

In future studies, it is worthwhile considering the excretion of fermentation products such as lactate to be reversible, as it was done in an earlier model^53^. To achieve that, a combination of that model and the one proposed in this article could be constructed. A further interesting extension is to consider the overall reaction from glutamine to pyruvate to be reversible. This would allow one to shed light on the energy balance of nitrogen storage and incorporation of glutamine into proteins. Another interesting point is that, for our model, the molecular masses of the enzymes were sufficient to predict a pathway’s cost, while other studies employ more elaborated formulations of pathway cost functions^42,45,58,71^. It is worth investigating whether this result of ours can be generalized. Such a suggestion could be corroborated by a study of the Warburg effect analog in *Escherichia coli*. In an earlier study of this so-called overflow metabolism, it was also pointed out that the preference of fermentation over respiration is a result of the latter pathway’s higher costs^72^.

In conclusion, our model is in good agreement with experimental observations of energy metabolism in eukaryotic cells. It shows that glutamine is an underestimated substrate for energy metabolism in comparison to glucose. Also it is versatile since it has a comparable ATP production rate per enzyme mass via respiration and fermentation and serves as a carbon as well as nitrogen source for biomass. The cross-species comparison disclosed that the optimal resource allocation in these metabolic models is influenced by the cost of the underlying pathways and is not uniform across the kingdoms of life. Our model can be applied by parameterization and stoichiometric adjustments to other species, including pathogens. The understanding of metabolic resource allocation is a crucial step for the identification of drug targets against microbial pathogens or cancer cells.

## Methods

### A minimal model to describe the Warburg and WarburQ effects

To achieve a model as small as possible, we lump entire pathways such as the tricarboxylic acid (TCA) cycle and oxidative phosphorylation into overall reactions (see Fig. 1), to describe the metabolic choices of a cell. We formalize a linear optimization problem of ATP generation under limited resources for enzyme synthesis. Reaction 1 represents the uptake of glucose as well as the upper part of glycolysis and reaction 4 represents the uptake of glutamine and its conversion to pyruvate (cf. Fig. 1A, B). Pyruvate is chosen as a central hub since it represents a branching point between fermentation (reaction 2, products like lactate or ethanol) and respiration (reaction 3, oxidation to CO_2_), and it is the only internal metabolite in this minimal model. Due to the addition of reaction 4 in comparison to earlier models, we explicitly consider the energy-rich cofactors NADH and FADH_2_ as external metabolites, which contribute to energy generation via the electron transport chain and ATPase by the conversion of ADP into ATP (reactions 5 and 6). *v*_1_–*v*_6_ denote the steady-state fluxes through the six aforementioned reactions. Like in previous models, we formulate ATP production rate as an objective function at a steady state, with the stoichiometric coefficients *m* of cofactor generation listed in Table 4. Only the stoichiometric coefficients *m*_ATP_ shape the objective function; the contribution of NADH and FADH_2_ to the energy balance is taken into account by considering the ETC in the model.

**Table 4:** Stoichiometric coefficients *m* of cofactor generation in reactions 1–6

		Reaction					
Cofactor	Coefficient	1	2	3	4	5	6
ATP	*m*_ATP_	2	0	1	1	2.5* or 1.5**	1.5
NADH	*m*_NADH_	2	−1	4	3	−1	0
FADH_2_	${m}_{FAD{H}_{2}}$	0	0	1	1	0	-1

1, glucose catabolism (up to pyruvate); 2, pyruvate fermentation; 3, pyruvate respiration; 4, glutamine catabolism (up to pyruvate); 5 and 6, reactions of the ETC.

*-for human and *Candida*-like models.^**^-for *S. cerevisiae* model.

In human cells, complex I of the ETC comprises 45 subunits and forms a supercomplex with other respiratory chain complexes, namely III and IV^73,74^. While in *S. cerevisiae*, the typical complex I is substituted by a smaller enzyme, encoded by the gene *NDI1*, which does not pump protons, *Candida albicans* and other pathogenic yeasts have both complex I and NDI1 analogs; this affects the ATP yield per NADH^75^. Accordingly, the three organisms show different ATP-over-glucose yields of respiration, notably 32 in *C. albicans*^20^, 16–18 in *S. cerevisiae*^12,76^, and about 30 in (healthy) humans^77^. Due to the immense difference between metabolisms of different cancer cell types, ATP yield in certain cancer cells may vary, but is assumed to be comparable to the value in healthy cells.

To account for the differences in ATP production, we compare three models, namely human cancer cells, *S. cerevisiae* with low-yield ETC and a *Candida*-like model with high-yield ETC and baker’s yeast enzyme masses (except for complex I). For humans and *C. albicans*, the following stoichiometric calculations can be done: one molecule of NADH starts a chain of reactions (beginning with complex I) that pump 10 H^+^, out of which 4 H^+^ are pumped by complex I itself^75,78^. 4 H^+^ are needed to produce 1 ATP, therefore the yield is 2.5 ATP per NADH (see Table 4). Since NDI1, which is *S. cerevisiae*’s substituent of complex I, does not pump protons, only 6 H^+^ are produced per NADH, so the yield is 1.5 ATP per NADH (Table 4)^75^.

As in previous models of the Warburg effect, we further assume a limit on the total flux sum (*C*), and the costs of fluxes are weighted with respect to their enzyme costs (*α*). By normalization, we set *C* = 1 and calculate *α*_*i*_ as enzyme mass (in MDa) per catalytic site across all reactions of the pathway (see Supplementary Tables 1 and 2). As a result, *J*_ATP_ can be interpreted as the amount of ATP per mole of substrate and per MDa of molecular enzyme mass required to catalyze the reactions. We used enzyme mass (molar mass based on amino acid sequence, as listed in UniProt^79^) as an estimation since it is available for enzymes with known amino acid sequence and has proven to be a good approximation of synthesis cost^45,58^. Further, the number of catalytic sites was inferred from PDB structures^80^ and published experimental studies (see Supplementary Tables 1, 2) to correctly account for the involved large enzyme complexes. The resulting enzyme masses per catalytic site and the corresponding cost factors *α*_*i*_ are listed in Table 5.

**Table 5:** Total enzyme mass per catalytic site of each overall reaction

Reaction	Cost factor	Human [MDa]	*S. cerevisiae* [MDa]	*Candida*-like [MDa]
1	*α*_1_	0.488	0.497	0.497
2	*α*_2_	0.037	0.098	0.098
3	*α*_3_	0.999	0.919	0.919
4	*α*_4_	0.671	0.862	0.862
5	*α*_5_	1.015	0.496	0.634
6	*α*_6_	0.755	0.434	0.434
all		3.964	3.306	3.444

For *Candida*-like species, a high-yield complex I of *C. albicans* is used instead of the low-yield but small *S. cerevisiae* variant. 1, glucose degradation (up to pyruvate); 2, pyruvate fermentation; 3, pyruvate respiration; 4, glutamine degradation (up to pyruvate); 5 and 6, reactions of ETC.

Our focus is on comparing glucose and glutamine as possible substrates rather than lactate or other fermentation products, which could be reabsorbed. For simplicity’s sake, we thus consider all reactions to be irreversible, so that the possible re-uptake of fermentation products^53^ is neglected.

In addition to the steady-state and cost constraints, we introduce a flux constraint in extension to the basic model. By modeling capacity constraints of certain reaction fluxes, we can mimic and study effects like the limited uptake rate of glucose or a saturation of enzymes like lactate dehydrogenase.

Overall, the resource allocation problem for the system under study (see Fig. 1) can be formally written as:

1 $${{Maximize}}\,(\,{{\mbox{ATP production rate}}})\quad {J}_{{{ATP}}}(v)=\mathop{\sum}\limits_{i}{m}_{{{ATP}}_{i}}\cdot {v}_{i}$$

2 $${{subject}}\,{{to}}\,(\,{{\mbox{steady state}}})\quad N\cdot v=0$$

3 $$\,{{\mbox{(cost constraint)}}}\,\quad \mathop{\sum}\limits_{i}{\alpha }_{i}\cdot {v}_{i}\le C$$

4 $$\,{{\mbox{(irreversibility)}}}\,\quad {v}_{i}\ge 0$$

5 $$\,{{\mbox{(flux constraint)}}}\,\quad {v}_{j}\le {v}_{cap,j}$$

where *v*_*i*_ is the flux (with the dimension [mM/s]) through the corresponding reaction *i*; ${m}_{AT{P}_{i}}$ is defined by the coefficients of ATP generation of the lumped reactions as described in Table 4 (first row).

In this formulation, a flux distribution of *v*_1−6_ is calculated, which maximizes the ATP production rate according to the mass stoichiometry coefficients *m* depicted in Fig. 1 and Table 4. This linear maximization of ATP production (equation (1)) is constrained by a steady-state assumption for internal metabolites (including energy-rich cofactors, equation (2)). Due to the required mass balance in a steady state, the system has three degrees of freedom, and the solution space can be visualized in a plot of *v*_1−3_ (Figs. 2A, 5), *v*_4_ being unambiguously defined by the rest of the carbon-transporting fluxes.

The sum of the fluxes *v*_*i*_ weighted by the protein mass coefficients *α* were limited by a cost constraint *C*, which is normalized to be unity (inequality (3)). Inequality (4) expresses our assumption that all reactions are irreversible. These constraints limit the feasible region to a closed polyhedron (see Fig. 2A). The last inequality (5) is taken into account only in an advanced model variant, where we include, in addition, an upper bound on the uptake rates of glucose and/or glutamine (see Figs. 4, 5).

Based on the human enzymes, homologous enzymes in *S. cerevisiae* were determined and differing reactions and non-homologous enzyme complexes are considered. Since enzyme masses of homologs are mostly comparable across eukaryotes, we simulate a *Candida*-like organism by using the enzyme masses from *S. cerevisiae* except the complex I, which is not present in this organism^73^.

### Model implementation

The linear optimization problem phrased in equations (1)–(5) was solved by the MATLAB routine linprog. The objective function was formulated as ATP production rate (1) divided by the cost (3). To simulate the optimal flux distribution for situations with additional constraints (5), a mesh of varying constraint values was generated in MATLAB, and linear optimization was performed for every node. The corresponding code can be found at https://git.uni-jena.de/qe45cow/warburq-minimal-model.

### Reporting summary

Further information on research design is available in the Nature Research Reporting Summary linked to this article.

### Supplementary information

Supplementary Material

Reporting Summary

## Data Availability

All data used are given in the manuscript and Supplementary information.

## Code availability

The computer code used for modeling can be found at https://git.uni-jena.de/qe45cow/warburq-minimal-model.

## References

[1] Rodríguez-Enríquez S, Marín-Hernández A, Gallardo-Pérez JC, Carreño-Fuentes L, Moreno-Sánchez R (2009). Targeting of cancer energy metabolism. Mol. Nutr. Food Res..

[2] Zhang Y, Yang J-M (2013). Altered energy metabolism in cancer: a unique opportunity for therapeutic intervention. Cancer Biol. Ther..

[3] Geeraerts X, Bolli E, Fendt S-M, Van Ginderachter JA (2017). Macrophage metabolism as therapeutic target for cancer, atherosclerosis, and obesity. Front. Immunol..

[4] Stavrum A-K, Heiland I, Schuster S, Puntervoll P, Ziegler M (2013). Model of tryptophan metabolism, readily scalable using tissue-specific gene expression data. J. Biol. Chem..

[5] Eisenreich W, Dandekar T, Heesemann J, Goebel W (2010). Carbon metabolism of intracellular bacterial pathogens and possible links to virulence. Nat. Rev. Microbiol..

[6] Ene IV, Brunke S, Brown AJP, Hube B (2014). Metabolism in fungal pathogenesis. Cold Spring Harb. Perspect. Med..

[7] Møller SH, Hsueh P-C, Yu Y-R, Zhang L, Ho P-C (2022). Metabolic programs tailor T cell immunity in viral infection, cancer, and aging. Cell Metab..

[8] Li S (2022). Metabolism drives macrophage heterogeneity in the tumor microenvironment. Cell Rep..

[9] Beuster G (2011). Inhibition of alanine aminotransferase in silico and in vivo promotes mitochondrial metabolism to impair malignant growth. J. Biol. Chem..

[10] Schuster S, Boley D, Möller P, Stark H, Kaleta C (2015). Mathematical models for explaining the Warburg effect: a review focussed on ATP and biomass production. Biochem. Soc. Trans..

[11] Stark H, Fichtner M, König R, Lorkowski S, Schuster S (2015). Causes of upregulation of glycolysis in lymphocytes upon stimulation. a comparison with other cell types. Biochimie.

[12] Pfeiffer T, Morley A (2014). An evolutionary perspective on the Crabtree effect. Front. Mol. Biosci..

[13] Warburg O, Minami S (1923). Versuche an überlebendem Carcinomgewebe. Klin. Wochenschr..

[14] Santos de Souza AC, Zenker Justo G, Ribeiro de Araújo D, Martins Cavagis AD (2011). Defining the molecular basis of tumor metabolism: a continuing challenge since Warburg’s discovery. Cell. Physiol. Biochem..

[15] Ganapathy-Kanniappan S, Geschwind J-FH (2013). Tumor glycolysis as a target for cancer therapy: progress and prospects. Mol. Cancer.

[16] Wang T, Ma F, Qian H-l (2021). Defueling the cancer: ATP synthase as an emerging target in cancer therapy. Mol. Ther. Oncolytics.

[17] De Deken R (1966). The Crabtree effect: a regulatory system in yeast. J. Gen. Microbiol..

[18] Askew C (2009). Transcriptional regulation of carbohydrate metabolism in the human pathogen *Candida albicans*. PLoS Pathog..

[19] Rozpedowska E (2011). *Candida albicans*–a pre-whole genome duplication yeast–is predominantly aerobic and a poor ethanol producer. FEMS Yeast Res..

[20] Ogasawara A (2006). Change in the respiration system of *Candida albicans* in the lag and log growth phase. Biol. Pharm. Bull..

[21] Sun N, Parrish RS, Calderone RA, Fonzi WA (2019). Unique, diverged, and conserved mitochondrial functions influencing *Candida albicans* respiration. mBio.

[22] Land GA, McDonald WC, Stjernholm RL, Friedman L (1975). Factors affecting filamentation in *Candida albicans*: changes in respiratory activity of *Candida albicans* during filamentation. Infect. Immun..

[23] Mulhern SM, Logue ME, Butler G (2006). *Candida albicans* transcription factor Ace2 regulates metabolism and is required for filamentation in hypoxic conditions. Eukaryot. Cell.

[24] Watanabe T, Ogasawara A, Mikami T, Matsumoto T (2006). Hyphal formation of *Candida albicans* is controlled by electron transfer system. Biochem. Biophys. Res. Commun..

[25] Bonhomme J (2011). Contribution of the glycolytic flux and hypoxia adaptation to efficient biofilm formation by *Candida albicans*. Mol. Microbiol..

[26] Noble SM, French S, Kohn LA, Chen V, Johnson AD (2010). Systematic screens of a *Candida albicans* homozygous deletion library decouple morphogenetic switching and pathogenicity. Nat. Genet..

[27] McDonough JA, Bhattacherjee V, Sadlon T, Hostetter MK (2002). Involvement of *Candida albicans* NADH dehydrogenase complex I in filamentation. Fungal Genet. Biol..

[28] Grahl N (2015). Mitochondrial activity and Cyr1 are key regulators of Ras1 activation of *C. albicans* virulence pathways. PLoS Pathog..

[29] Karinch AM, Pan M, Lin C-M, Strange R, Souba WW (2001). Glutamine metabolism in sepsis and infection. J. Nutr..

[30] Scalise M, Pochini L, Galluccio M, Indiveri C (2016). Glutamine transport. From energy supply to sensing and beyond. Biochim. Biophys. Acta.

[31] Tildón, J. T. & Zielke, H. R. Glutamine: an energy source for mammalian tissues. In *Glutamine and Glutamate Mammals*, Vol. I (ed. Kvamme, E.) 167–182 (CRC Press, 2018).

[32] Garbe E, Vylkova S (2019). Role of amino acid metabolism in the virulence of human pathogenic fungi. Curr. Clin. Microbiol. Rep..

[33] McKeehan WL (1982). Glycolysis, glutaminolysis and cell proliferation. Cell Biol. Int. Rep..

[34] Jin L, Alesi GN, Kang S (2016). Glutaminolysis as a target for cancer therapy. Oncogene.

[35] Damiani C (2017). A metabolic core model elucidates how enhanced utilization of glucose and glutamine, with enhanced glutamine-dependent lactate production, promotes cancer cell growth: The WarburQ effect. PLoS Comput. Biol..

[36] Dang CV (2010). Glutaminolysis: supplying carbon or nitrogen or both for cancer cells?. Cell Cycle.

[37] Güemes M, Rahman SA, Hussain K (2016). What is a normal blood glucose?. Arch. Dis. Child..

[38] Brosnan JT (2003). Interorgan amino acid transport and its regulation. J. Nutr..

[39] Pfeiffer T, Schuster S, Bonhoeffer S (2001). Cooperation and competition in the evolution of ATP-producing pathways. Science.

[40] Schuster S, de Figueiredo LF, Schroeter A, Kaleta C (2011). Combining metabolic pathway analysis with evolutionary game theory. explaining the occurrence of low-yield pathways by an analytic optimization approach. Biosystems.

[41] Vazquez A, Oltvai ZN (2011). Molecular crowding defines a common origin for the Warburg effect in proliferating cells and the lactate threshold in muscle physiology. PLoS ONE.

[42] Shlomi T, Benyamini T, Gottlieb E, Sharan R, Ruppin E (2011). Genome-scale metabolic modeling elucidates the role of proliferative adaptation in causing the Warburg effect. PLoS Comput. Biol..

[43] Parker GA, Smith JM (1990). Optimality theory in evolutionary biology. Nature.

[44] Dühring S (2017). Modelling the host–pathogen interactions of macrophages and *Candida albicans* using game theory and dynamic optimization. J. R. Soc. Interface.

[45] Noor E (2016). The protein cost of metabolic fluxes: prediction from enzymatic rate laws and cost minimization. PLoS Comput. Biol..

[46] Famili I, Förster J, Nielsen J, Palsson BO (2003). *Saccharomyces cerevisiae* phenotypes can be predicted by using constraint-based analysis of a genome-scale reconstructed metabolic network. Proc. Natl Acad. Sci. USA.

[47] Schuster S, Pfeiffer T, Fell DA (2008). Is maximization of molar yield in metabolic networks favoured by evolution?. J. Theor. Biol..

[48] Warburg O (1956). On the origin of cancer cells. Science.

[49] Resendis-Antonio O, Checa A, Encarnación S (2010). Modeling core metabolism in cancer cells: surveying the topology underlying the Warburg effect. PLoS ONE.

[50] Müller S, Regensburger G, Steuer R (2014). Enzyme allocation problems in kinetic metabolic networks: optimal solutions are elementary flux modes. J. Theor. Biol..

[51] Schuster S, Boley D, Möller P, Kaleta C (2015). A minimal model for explaining the higher ATP production in the Warburg effect. PeerJ Prepr..

[52] Dai Z, Shestov AA, Lai L, Locasale JW (2016). A flux balance of glucose metabolism clarifies the requirements of the Warburg effect. Biophys. J..

[53] Möller P, Liu X, Schuster S, Boley D (2018). Linear programming model can explain respiration of fermentation products. PLoS ONE.

[54] Guigas G, Weiss M (2016). Effects of protein crowding on membrane systems. Biochim. Biophys. Acta.

[55] Schlame M (2021). Protein crowding in the inner mitochondrial membrane. Biochim. Biophys. Acta.

[56] Mazat J-P, Ransac S (2019). The fate of glutamine in human metabolism. Metabolites.

[57] Schuster S, Fell DA, Dandekar T (2000). A general definition of metabolic pathways useful for systematic organization and analysis of complex metabolic networks. Nat. Biotechnol..

[58] Wortel MT, Noor E, Ferris M, Bruggeman FJ, Liebermeister W (2018). Metabolic enzyme cost explains variable trade-offs between microbial growth rate and yield. PLoS Comput. Biol..

[59] Guillamón JM, van Riel NA, Giuseppin ML, Verrips CT (2001). The glutamate synthase (GOGAT) of *Saccharomyces cerevisiae* plays an important role in central nitrogen metabolism. FEMS Yeast Res..

[60] Olivares-Marin IK, González-Hernández JC, Regalado-Gonzalez C, Madrigal-Perez LA (2018). *Saccharomyces cerevisiae* exponential growth kinetics in batch culture to analyze respiratory and fermentative metabolism. J. Vis. Exp..

[61] Schwartz L, T Supuran C, O Alfarouk K (2017). The Warburg effect and the hallmarks of cancer. Anticancer Agents Med. Chem..

[62] Bergers G, Fendt S-M (2021). The metabolism of cancer cells during metastasis. Nat. Rev. Cancer.

[63] Wortel MT, Peters H, Hulshof J, Teusink B, Bruggeman FJ (2014). Metabolic states with maximal specific rate carry flux through an elementary flux mode. FEBS J..

[64] Soberón M, Olamendi J, Rodriguez L, Gonzalez A (1989). Role of glutamine aminotransferase in glutamine catabolism by *Saccharomyces cerevisiae* under microaerophilic conditions. J. Gen. Microbiol..

[65] Ter Schure EG (1998). Repression of nitrogen catabolic genes by ammonia and glutamine in nitrogen-limited continuous cultures of *Saccharomyces cerevisiae*. Microbiology.

[66] Sahu U, Rangarajan PN (2016). Methanol expression regulator 1 (Mxr1p) is essential for the utilization of amino acids as the sole source of carbon by the methylotrophic yeast, *Pichia pastoris*. J. Biol. Chem..

[67] Zhu H (2023). Effect of DAP and glutamine supplementation on sulfur-containing volatiles and sensory properties of Chardonnay wine fermented with *Saccharomyces cerevisiae* yeast. J. Food Sci..

[68] Vander Heiden MG, Cantley LC, Thompson CB (2009). Understanding the Warburg effect: the metabolic requirements of cell proliferation. Science.

[69] Imran, M. et al. Monitoring of glucose, salt and pure water in human whole blood: an in vitro study. *Pak. J. Pharm. Sci.* **29**, 1237–1242 (2016). 27393437

[70] Fan J (2013). Glutamine-driven oxidative phosphorylation is a major ATP source in transformed mammalian cells in both normoxia and hypoxia. Mol. Syst. Biol..

[71] Flamholz A, Noor E, Bar-Even A, Liebermeister W, Milo R (2013). Glycolytic strategy as a tradeoff between energy yield and protein cost. Proc. Natl Acad. Sci. USA.

[72] Basan M (2015). Overflow metabolism in *Escherichia coli* results from efficient proteome allocation. Nature.

[73] Schägger H, Pfeiffer K (2000). Supercomplexes in the respiratory chains of yeast and mammalian mitochondria. EMBO J..

[74] Stroud DA (2016). Accessory subunits are integral for assembly and function of human mitochondrial complex I. Nature.

[75] Li D, She X, Calderone R (2016). Functional diversity of complex I subunits in *Candida albicans* mitochondria. Curr. Genet..

[76] Verduyn C, Stouthamer AH, Scheffers WA, van Dijken JP (1991). A theoretical evaluation of growth yields of yeasts. Antonie Van Leeuwenhoek.

[77] Rich P (2003). The molecular machinery of Keilin’s respiratory chain. Biochem. Soc. Trans..

[78] Hirst J (2013). Mitochondrial complex I. Annu. Rev. Biochem..

[79] UniProt Consortium. (2018). UniProt: the universal protein knowledgebase. Nucleic Acids Res..

[80] Burley SK (2018). RCSB protein data bank: sustaining a living digital data resource that enables breakthroughs in scientific research and biomedical education. Protein Sci..

[81] Ortmayr K, Dubuis S, Zampieri M (2019). Metabolic profiling of cancer cells reveals genome-wide crosstalk between transcriptional regulators and metabolism. Nat. Commun..

[82] Chitu V, Yeung Y-G, Yu W, Nandi S, Stanley ER (2011). Measurement of macrophage growth and differentiation. Curr. Protoc. Immunol..

[83] Italiani P, Boraschi D (2014). From monocytes to M1/M2 macrophages: phenotypical vs. functional differentiation. Front. Immunol..

[84] André LM, Ausems CRM, Wansink DG, Wieringa B (2018). Abnormalities in skeletal muscle myogenesis, growth, and regeneration in myotonic dystrophy. Front. Neurol..

[85] Salari R, Salari R (2017). Investigation of the best *Saccharomyces cerevisiae* growth condition. Electron. Physician.

[86] Alloue-Boraud WAM (2015). Fermentation profile of *Saccharomyces cerevisiae* and *Candida tropicalis* as starter cultures on barley malt medium. J. Food Sci. Technol..

[87] Kochi Y, Matsumoto Y, Sekimizu K, Kaito C (2017). Two-spotted cricket as an animal infection model of human pathogenic fungi. Drug Discov. Ther..

[88] Hargreaves, M. & Spriet, L. L. Skeletal muscle energy metabolism during exercise. *Nat. Metab.* **2**, 817–828 (2020). 10.1038/s42255-020-0251-4 32747792

[89] Tucey TM (2018). Glucose homeostasis is important for immune cell viability during *Candida* challenge and host survival of systemic fungal infection. Cell Metab..

[90] Kelly B, O'Neill LAJ (2015). Metabolic reprogramming in macrophages and dendritic cells in innate immunity. Cell Res..

[91] Yajima D (2006). Ethanol production by *Candida albicans* in postmortem human blood samples: effects of blood glucose level and dilution. Forensic Sci. Int..

