# Supplementary material for: Including glutamine in a resource allocation model of energy metabolism in cancer and yeast cells
Source: NPJ Syst Biol Appl. 2024 Jul 18;10:77. doi: 10.1038/s41540-024-00393-x (PMC11258256; doi:10.1038/s41540-024-00393-x)
Supplement: Supplementary file 1 — Supplementary Material [file 41540_2024_393_MOESM1_ESM.pdf]

Supplementary Table 1

List of human enzymes catalyzing each reaction of the lumped overall reactions 1-6. Includes UniProt IDs of subunits and references for subunit compositions as well as number of catalytic sites to infer cost factors α_{1-6}.

Pathways

Pathway	EC Number	Name	Uniprot ID(s)	AA length	Mass (Da)	Subunits	Catalytic sites	Reference	Mass per catalytic site (Da)	Pathway sum (Da)	Average mass per reaction
**v1** (Glycolysis)	**2.7.1.1**	Hexokinase-1	P19367	917	102.486	1	2	PDB: 1DGK	51.243		
	**5.3.1.9**	Glucose-6-phosphate isomerase	P06744	558	63.147	2	2	PDB: 1HOX	63.147		
	**2.7.1.11**	ATP-dependent 6-phosphofructokinase	P08237	780	85.183	4	4	PDB: 4PFK	85.183		
	**4.1.2.13**	Fructose-bisphosphate aldolase B	P05062	364	39.473	4	4	PDB: 4ALD, 1QO5	39.473		
	**5.3.1.1**	Triosephosphate isomerase	P60174	286	30.791	2	2	PDB: 1HTI	30.791		
	**1.2.1.12**	Glyceraldehyde-3-phosphate dehydrogenase	P04406	335	36.053	4	4	PDB: 3GPD	36.053		
	**2.7.2.3**	Phosphoglycerate kinase 1	P00558	417	44.615	1	1	PDB: 3C39	44.615		
	**5.4.2.11**	Phosphoglycerate mutase 1	P18669	254	28.804	2	2	PDB: 1YFK	28.804		
	**4.2.1.11**	Enolase 1	P06733	434	47.169	2	2	PDB: 3B97	47.169		
	**2.7.1.40**	Pyruvate kinase PKLR	P30613	574	61.830	4	4	PDB: 2VGB	61.830		
										**488.308**	48.831
**v2** (Lac ferm)	**1.1.1.27**	L-lactate dehydrogenase	P00338	332	36.689	4	4	PDB: 1I10	36.689		
										**36.689**	36.689
**v3** (PDH)	**1.2.4.1**	E1 PDHA1	P08559	390	43.296	2	2	https://doi.org/10.1074/jbc.m300339200	82.529		

		E1 PDHB	P11177	359	39.233	2					
	**2.3.1.12***	E2 Dihydrolipoyllysine-residue acetyltransferase	P10515	647	68.997	40	30	PDB: 6H55	128.077		
		E3 binding protein	O00330	501	54.122	20		PDB: 6H55			
	**1.8.1.4**	E3 Dihydrolipoyl dehydrogenase	P09622	509	54.177	2	2	PDB: 5NHG	54.177		
										**264.783**	88.261
**v3** (TCA)	**2.3.3.1**	citrate synthase	O75390	466	51.712	2	2	PDB: 5UZP	51.712		
	**4.2.1.3**	aconitate hydratase	Q99798	780	85.425	1	1	PDB: 2B3Y	85.425		
	**1.1.1.41****	Isocitrate dehydrogenase [NAD] alpha	P50213	366	39.592	2	2	https://doi.org/10.1038/srep41882	82.081		
		Isocitrate dehydrogenase [NAD] beta	O43837	385	42.184	1					
		Isocitrate dehydrogenase [NAD] gamma	P51553	393	42.794	1					
	**1.2.4.2**	2-Oxoglutarate Dehydrogenase Complex (E1)	Q02218	1023	115.935	2	2	PDB: 2JGD	115.935		
	**2.3.1.61**	2-Oxoglutarate Dehydrogenase Complex (E2)	P36957	453	48.755	24	24	https://doi.org/10.1006/jmbi.1998.1924	48.755		
	**1.8.1.4**	2-Oxoglutarate Dehydrogenase Complex (E3)	P09622	509	54.177	2	2	PDB: 5J5Z	54.177		
	**6.2.1.4**	Succinate--CoA ligase alpha	P53597	346	36.250	1			82.761		
		Succinate--CoA ligase beta	Q96I99	432	46.511	1	1	PDB: 2FP4			
	**1.3.5.1**	Succinate dehydrogenase A	P31040	664	72.692	1	1	PDB: 3SFD	122.932		

		Succinate dehydrogenase B	P21912	280	31.630	1					
		Succinate dehydrogenase C	Q99643	169	18.610	1					
		Succinate dehydrogenase D	O14521	159	17.043	1					
	**4.2.1.2**	Fumarate hydratase	P07954	510	54.637	4	4	PDB: 5UPP	54.637		
	**1.1.1.37**	Malate dehydrogenase	P40926	338	35.503	2	2	PDB: 2DFD	35.503		
										**733.918**	73.392

Comments for v3:
* - https://doi.org/10.1016/j.str.2019.04.009 “This structural arrangement is capable of yielding up to 30 active acetyltransferase sites (10 E23 trimers + 10 E21-E3BP2 trimers).1*2”
****** - beta and gamma subunits have regulatory roles

**v4** (Gln)	**3.5.1.2**	Glutaminase	Q9UI32	602	66.323	4	4	PDB:3UNW	66.323		
	**1.4.1.3**	Glutamate dehydrogenase	P00367	558	61.398	4	4	PDB: 6DHD	61.398		
	**1.2.4.2**	2-Oxoglutarate Dehydrogenase Complex (E1)	Q02218	1023	115.935	2	2	PDB: 2JGD	115.935		
	**2.3.1.61**	2-Oxoglutarate Dehydrogenase Complex (E2)	P36957	453	48.755	24	24	https://doi.org/10.1006/jmbi.1998.1924	48.755		
	**1.8.1.4**	2-Oxoglutarate Dehydrogenase Complex (E3)	P09622	509	54.177	2	2	PDB: 5J5Z	54.177		
	**6.2.1.4**	Succinate--CoA ligase alpha	P53597	346	36.250	1			82.761		
		Succinate--CoA ligase beta	Q96I99	432	46.511	1	1	PDB: 2FP4			
	**1.3.5.1**	Succinate dehydrogenase A	P31040	664	72.692	1	1	PDB: 3SFD	122.932		
		Succinate dehydrogenase B	P21912	280	31.630	1					
		Succinate dehydrogenase C	Q99643	169	18.610	1					

		Succinate dehydrogenase D	O14521	159	17.043	1					
	**4.2.1.2**	Fumarate hydratase	P07954	510	54.637	4	4	PDB: 5UPP	54.637		
	**1.1.1.40**	NADP-dependent malic enzyme	P48163	572	64.150	4	4	PDB: 1GQ2	64.150		
										**671.068**	74.563
**v5** (oxPho NADH)	**1.6.99.3**	Complex I			1.043.099	1	4	1MDa confirmed here: https://doi.org/10.1074/jbc.m607135200	260.775		
	**1.10.2.2**	Complex III			265.001	1	2	same range as in PDB: 1NTZ	132.501		
	**1.9.3.1**	Complex IV			319.236	1	2	comparable to PDB structures like: 2EIJ	159.618		
	**3.6.3.14**	ATP Synthase			1.387.419	1	3		462.473		
										**1.015.366**	253.842
**Comments for v5:** complex II is not required											
**v6** (oxPho FADH2)	**1.3.5.1**	Complex II			122.932	1	2	see TCA cycle	0		
	**1.10.2.2**	Complex III			265.001	1	2	same range as in PDB: 1NTZ	132.501		
	**1.9.3.1**	Complex IV			319.236	1	2	comparable to PDB structures like: 2EIJ	159.618		
	**3.6.3.14**	ATP Synthase			1.387.419	1	3		462.473		
										**754.592**	188.648
**Comments for v6:** complex I is not required; complex II is already present in v3 and v4											

Alphas

Overall reaction	Mass in Da	alpha	
1	488.308	α1	0,4883
2	36.689	α2	0,0367
3	998.701	α3	0,9987
4	671.068	α4	0,6711
5	1.015.366	α5	1,0154
6	754.592	α6	0,7546
all	3.964.724		3,9647

Supplementary Table 2

List of yeast enzymes catalyzing each reaction of the lumped overall reactions 1-6. Includes UniProt IDs of subunits and references for subunit compositions as well as number of catalytic sites to infer cost factors $\alpha_{1\text{-}6}$.

Pathways

Pathway	EC Number	Name	Uniprot ID(s)	AA length	Mass (Da)	Subunits	Catalytic sites	Reference	Mass per catalytic site (Da)	Pathway sum (Da)	Average mass per reaction
**v1** (Glycolysis)	**2.7.1.1**	Hexokinase-1	P04806	485	53.738	1	1	PDB: 3B8A	53.738		
	**5.3.1.9***	Glucose-6-phosphate isomerase	P12709	554	61.299	2	2	PDB: 1HOX	61.299		
	**2.7.1.11**	ATP-dependent 6-phosphofructokinase subunit alpha	P16861	987	107.970	4	4	PDB: 3O8O	106.294		
		ATP-dependent 6-phosphofructokinase subunit beta	P16862	959	104.618	4	4				
	**4.1.2.13****	Fructose-bisphosphate aldolase	P14540	359	39.621	4	4	PDB: 1ZEN	39.621		
	**5.3.1.1**	Triosephosphate isomerase	P00942	248	26.795	2	2	PDB: 1I45	26.795		
	**1.2.1.12**	Glyceraldehyde-3-phosphate dehydrogenase	P00359	332	35.747	4	4	PDB: 3PYM	35.747		
	**2.7.2.3**	Phosphoglycerate kinase 1	P00560	416	44.738	1	1	PDB: 1FW8	44.738		
	**5.4.2.11**	Phosphoglycerate mutase 1	P00950	247	27.609	4	4	PDB: 1BQ3	27.609		
	**4.2.1.11**	Enolase 1	P00924	437	46.816	2	2	PDB: 2ONE	46.816		
	**2.7.1.40**	Pyruvate kinase 1	P00549	500	54.545	4	4	PDB: 1A3W	54.545		
										**497.202**	49.720

**Comments for v1:**<br>***** - no PDB Entry for yeast.<br>****** - Fructose bisphosphate aldolase is involved in the control of RNA polymerase III-directed transcription. https://doi.org/10.1016/j.bbamcr.2014.02.007											
**v2** (Eth ferm)	**4.1.1.1***	Pyruvate decarboxylase 1	P06169	563	61.495	4	4	PDB: 1PYD	61.495		
	**1.1.1.1****	Alcohol dehydrogenase I	P00330	348	36.849	4	4	PDB: 4W6Z	36.849		
										**98.344**	98.344
**Comments for v2:**<br>***** - Pyruvate decarboxylases 1, 2 and 3 are isoenzymes, PDC1 under normal conditions (https://biocyc.org/META/new-image?type=PATHWAY&object=PWY-5486&detail-level=2&ENZORG=TAX-4932)<br>****** - ADH1 main isoform for fermentation											
**v3** (PDH)	**1.2.4.1**	PDA1	P16387	420	46.343	2	2	Uniprot P16387 “Pyruvate dehydrogenase (E1) is a tetramer of 2 alpha and 2 beta subunits”	86.397		
		PDB1	P32473	366	40.054	2					
	**2.3.1.12***	E2 Dihydrolipoyllysine-residue acetyltransferase	P12695	482	51.818	40	30		99.332		
		E3 binding protein	P16451	410	45.362	20					
	**1.8.1.4**	E3 Dihydrolipoyl dehydrogenase	P09624	499	54.010	2	2	PDB: 1JEH	54.010		
										**239.739**	79.913
**v3** (TCA)	**2.3.3.1**	citrate synthase	P00890	479	53.360	2	2		53.360		
	**4.2.1.3**	aconitate hydratase	P19414	778	85.368	1	1		85.368		
	**1.1.1.41****	Isocitrate dehydrogenase [NAD] alpha	P28834	360	39.324	2	2	PDB: 3BLW	79.063		
		Isocitrate dehydrogenase [NAD] beta	P28241	369	39.739	2	0	regulatory sites in IDH1			
	**1.2.4.2**	2-Oxoglutarate Dehydrogenase Complex (E1)	P20967	1014	114.416	2	2		114.416		

	**2.3.1.61**	2-Oxoglutarate Dehydrogenase Complex (E2)	P19262	463	50.431	24	24	https://doi.org/10.1006/jmbi.1998.1924	50.431		
	**1.8.1.4**	2-Oxoglutarate Dehydrogenase Complex (E3)	P09624	499	54.010	2	2	PDB: 1JEH	54.010		
	**6.2.1.4*****	Succinate--CoA ligase alpha	P53598	329	35.032	1			81.933		
		Succinate--CoA ligase beta	P53312	427	46.901	1	1				
	**1.3.5.1**	Succinate dehydrogenase A	Q00711	640	70.229	1	2	PDB: 1NEK	71.389		
		Succinate dehydrogenase B	P21801	266	30.231	1					
		Succinate dehydrogenase C	P33421	198	22.068	1					
		Succinate dehydrogenase D	P37298	181	20.249	1					
	**4.2.1.2**	Fumarate hydratase	P08417	488	53.152	4	4	PDB: 1YFM	53.152		
	**1.1.1.37*******	Malate dehydrogenase	P17505	334	35.650	2	2	PDB: 2DFD	35.650		
										**678.772**	67.877

Comments for v3:
***** - PDH Complex of yeast is similar to mammalian PDH complex: https://doi.org/10.1111/j.1432-1033.1981.tb05647.x
****** - Tetramer : http://pdb101.rcsb.org/motm/129 ; no gamma subunit found for yeast
******* - LSC1 & LSC2
******** - MDH1, mitochondrial isoenzyme (TCA cycle)

**v4** (Gln)	**1.4.1.13***	Glutamate synthase	Q12680	2145	238.102	3	3	https://swissmodel.expasy.org/repository/uniprot/Q12680?csm=78184877D2167A0D	238.102		
	**1.4.1.2****	Glutamate dehydrogenase	P33327	1092	124.332	1	1	https://biocyc.org/META/NEW-IMAGE?type=PATHWAY&object=GLUTAMATE-DEG1-PWY	124.332		

	**1.2.4.2**	2-Oxoglutarate Dehydrogenase Complex (E1)	P20967	1014	114.416	2	2		114.416		
	**2.3.1.61**	2-Oxoglutarate Dehydrogenase Complex (E2)	P19262	463	50.431	24	24	https://doi.org/10.1006/jmbi.1998.1924	50.431		
	**1.8.1.4**	2-Oxoglutarate Dehydrogenase Complex (E3)	P09624	499	54.010	2	2	PDB: 1JEH	54.010		
	**6.2.1.4**	Succinate--CoA ligase alpha	P53598	329	35.032	1	1		81.933		
		Succinate--CoA ligase beta	P53312	427	46.901	1		PDB: 2FP4			
	**1.3.5.1**	Succinate dehydrogenase A	Q00711	640	70.229	1	2	PDB: 1ORZ	71.389		
		Succinate dehydrogenase B	P21801	266	30.231	1					
		Succinate dehydrogenase C	P33421	198	22.068	1					
		Succinate dehydrogenase D	P37298	181	20.249	1					
	**4.2.1.2**	Fumarate hydratase	P08417	488	53.152	4	4	PDB: 1YFM	53.152		
	**1.1.1.40**	NADP-dependent malic enzyme	P36013	669	74.376	4	4	PDB: 1GQ2	74.376		
										**862.141**	95.793

Comments for v4:
* - GOGAT system is alternative route for glutamine uptake
** - The main pathway for Saccharomyces cerevisiae glutamate degradation is catalyzed by the NAD-dependent glutamate dehydrogenase encoded by GDH2
https://biocyc.org/META/NEW-IMAGE?type=PATHWAY&object=GLUTAMATE-DEG1-PWY

**v5** (oxPho NADH)	**1.6.99.3***	External NADH-ubiquinone oxidoreductase 1, mitochondrial	P40215	560	62.774	2	2		62.217		

**1.6.99.3**	External NADH-ubiquinone oxidoreductase 2, mitochondrial	Q07500	545	61.659	2	2				
**7.1.1.2**	Complex I			800.000		4	PMC4724564	200.000		
**1.10.2.2****	QCR10	P37299	77	8.593	1	2	PDB: 1EZV			
	QCR9	P22289	66	7.476	1					
	QCR8	P08525	94	10.975	1					
	QCR7	P00128	127	14.565	1					
	QCR6	P00127	147	17.257	1					
	QCR2	P07257	368	40.478	1					
	COR1	P07256	457	50.228	1					
	CYT1	P07143	309	34.055	1					
	RIP1	P08067	215	23.365	1					
	COB	P00163	385	43.656	1			125.324		
**1.9.3.1*****	COX1	P00401	534	58.798	1	2	PDB: 5IY5			
	COX12	Q01519	83	9.788	1					
	COX13	P32799	129	15.021	1					
	COX2	P00410	251	28.567	1					
	COX3	P00420	269	30.360	1					
	COX4	P04037	155	17.143	1					
	COX5A	P00424	153	17.140	1					
	COX6	P00427	148	17.342	1					
	COX7	P10174	60	6.932	1					
	COX8	P04039	78	8.907	1					
	COX9	P07255	59	6.963	1			108.481		
**3.6.3.14**	ATP Synthase			600.000	17	3	https://www.pnas.org/content/109/34/13602	200.000		
									**496.021**	139.204

**Comments for v5:**<br>***** - In Saccharomyces cerevisiae, complex I is replaced by type II NADH dehydrogenases, which are peripheral dimeric membrane proteins:<br>****** - https://pathway.yeastgenome.org/YEAST/NEW-IMAGE?type=REACTION-IN-PATHWAY&object=RXN3O-168<br>******* - There is a single- and a double-catalytic site model https://www.embopress.org/doi/full/10.15252/embj.201695021.											
**v6** (oxPho FADH2)	**1.3.5.1**	Complex II			70.229	1	2	see TCA cycle	0		
	**1.10.2.2***	Complex III			250.648	1	2	same range as in PDB: 1NTZ	125.324		
	**1.9.3.1****	Complex IV			216.961	1	2	comparable to PDB structures like: 2EIJ	108.481		
	**3.6.3.14**	ATP Synthase			600.000	1	3	https://www.pnas.org/content/109/34/13602	200.000		
										**433.805**	108.451
**Comments for v6:**<br>Complex I is not required; complex II is already present in v3 and v4<br>***** and ****** - more detailed in v5											

Alphas

S. cerevisiae

Overall reaction	Mass in Da	alpha	
v1	497.202	α1	0,497202
v2	98.344	α2	0,098344
v3	918.511	α3	0,9185105
v4	862.141	α4	0,8621405
v5	496.021	α5	0,496021
v6	433.805	α6	0,4338045
all	3.306.023		3,3060225

Candida- like (with complex I)

Overall reaction	Mass in Da	alpha	
v1	497.202	α1	0,4972
v2	98.344	α2	0,0983
v3	918.511	α3	0,9185
v4	862.141	α4	0,8621
v5	633.805	α5	0,6338
v6	433.805	α6	0,4338
all	3.443.806		3,4438
